# P-939. 2-year Follow-up on Patients Who Inject Drugs (PWID) Diagnosed with Infective Endocarditis (IE)

**DOI:** 10.1093/ofid/ofae631.1130

**Published:** 2025-01-29

**Authors:** Kaya Patel, Olivia Duffield, Sara K Schultz, Stephanie Spivack

**Affiliations:** Perelman School of Medicine , Philadelphia, Pennsylvania; Temple University School of Medicine, Philadelphia, Pennsylvania; Temple University Hospital, Philadelphia, Pennsylvania; Temple University Health System, Philadelphia, Pennsylvania

## Abstract

**Background:**

IE is a serious infectious complication of intravenous drug use (IVDU). Treatments include prolonged courses of IV antibiotics, surgical valve repair or replacement, and vacuum-assisted thrombectomy. Herein, we describe a 2-year follow up of outcomes of PWID who presented with IE.

**Figure:**
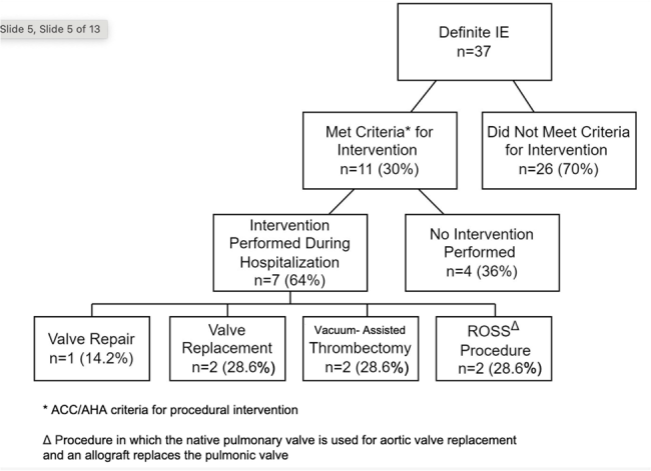

Treatment modalities for IE

**Methods:**

The IRB approved this study. We queried our EMR for patients with documented opioid use disorder (OUD) with injection behavior and positive blood cultures from September 2021 to March 2022. For patients identified to have IE in this time frame, we conducted a retrospective chart review from March 2022- March 2024 to evaluate outcomes.

**Figure:**
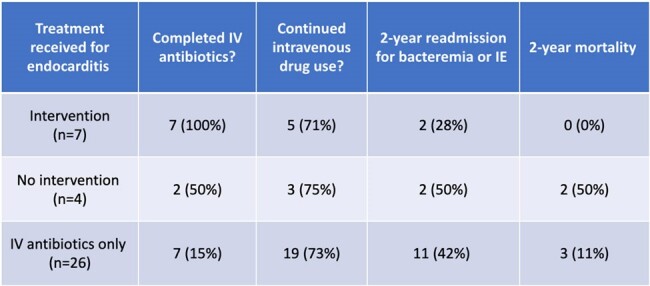

Outcomes in all treatment groups

**Results:**

We identified 37 patients with definite IE by modified Duke’s criteria. The treatment modalities these patients received are described in Figure 1. Of the 11 patients meeting criteria to receive surgical intervention, only 7 (63%) of these patients received intervention. All 7 (100%) of the patients that received intervention completed their antibiotic course for treatment. None were readmitted for sequela of undertreated endocarditis, but 2 (28%) patients were readmitted within 2 years for repeat endocarditis due to continued injection drug use. The 2-year all-cause mortality in this group was 0%. Of the 4 (37%) patients who required intervention but did not receive it, 1 (25%) was offered intervention but declined, 1 (25%) had an in-hospital death, and 2 (50%) left with a patient directed discharge but received intervention when readmitted within 3 months. The 2-year all-cause mortality rate in this group was 50%.

In the remaining 26 patients who did not require intervention, 7 (27%) completed an antibiotic treatment course, 17 (65%) did not complete antibiotics, and 2 (8%) had an unknown treatment course. Eleven (42%) were readmitted for bacteremia within 2 years. Four (15%) were readmitted with endocarditis (3 within 60 days from untreated endocarditis with worsening vegetation on TTE and 1 with endocarditis from continued injection drug use). The 2-year all-cause mortality in this group was 3 (11%). Table 1.

**Conclusion:**

In a 2-year follow-up of PWID presenting with definite endocarditis, high survival was noted in patients who underwent intervention. In patients receiving antibiotic therapy alone, high rates of incomplete treatment and readmissions occurred.

**Disclosures:**

**Sara K. Schultz, MD FACP FIDSA**, AbbVie: Advisor/Consultant

